# Impact of Seminal Plasma Antioxidants on Donkey Sperm Cryotolerance

**DOI:** 10.3390/antiox11020417

**Published:** 2022-02-18

**Authors:** Jaime Catalán, Iván Yánez-Ortiz, Asta Tvarijonaviciute, Luis Guillermo González-Arostegui, Camila P. Rubio, Marc Yeste, Jordi Miró, Isabel Barranco

**Affiliations:** 1 Equine Reproduction Service, Department of Animal Medicine and Surgery, Faculty of Veterinary Sciences, Autonomous University of Barcelona, ES-08193 Cerdanyola del Vallès, Spain; dr.jcatalan@gmail.com (J.C.); ivan.yanez22@gmail.com (I.Y.-O.); 2 Biotechnology of Animal and Human Reproduction (TechnoSperm), Institute of Food and Agricultural Technology, University of Girona, ES-17003 Girona, Spain; marc.yeste@udg.edu; 3 Unit of Cell Biology, Department of Biology, Faculty of Sciences, University of Girona, E-17003 Girona, Spain; 4 Faculty of Veterinary Medicine, University of Teramo, Loc. Piano d’Accio, IT-64100 Teramo, Italy; 5 Department of Medicine and Animal Surgery, Faculty of Veterinary Medicine, University of Murcia, ES-30100 Murcia, Spain; asta@um.es (A.T.); luisgarostegui@gmail.com (L.G.G.-A.); 6 Interdisciplinary Laboratory of Clinical Analysis Interlab-UMU, Faculty of Veterinary Medicine, Regional Campus of International Excellence ‘Campus Mare Nostrum’, University of Murcia, ES-30100 Murcia, Spain; camila.peres@uab.cat; 7 Department of Animal and Food Science, Faculty of Veterinary Sciences, Autonomous University of Barcelona, ES-08193 Cerdanyola del Vallès, Spain; 8 Department of Veterinary Medical Sciences, University of Bologna, IT-40064 Ozzano dell’Emilia, Italy

**Keywords:** seminal plasma (SP), sperm, donkey, cryopreservation, antioxidants, reactive oxygen species (ROS)

## Abstract

This study investigated whether the activities of the antioxidant components of donkey seminal plasma (SP)—both enzymatic (superoxide dismutase (SOD), catalase-like (CAT), glutathione peroxidase-like (GPX), and paraoxonase type 1 (PON1)) and non-enzymatic (measured in terms of total thiol, copper-reducing antioxidant capacity (CUPRAC), ferric-reducing ability of plasma (FRAP), and Trolox equivalent antioxidant capacity (TEAC))—and oxidative stress index (OSI) are related to sperm cryotolerance. For this purpose, 15 ejaculates from jackasses (one per individual) were collected and split into two aliquots. The first one was used for measuring the activities levels of enzymatic and non-enzymatic antioxidants and OSI in SP, whereas the other aliquot was cryopreserved. Before cryopreservation, sperm quality parameters (concentration, motility, and viability) were evaluated. After thawing, sperm motility, plasma membrane integrity, lipid disorder, mitochondrial membrane potential, reactive oxygen species (ROS), and calcium intracellular levels were also determined. Based on the percentages of total motility (TM) and of sperm with an intact plasma membrane (SYBR14^+^/PI^−^) after thawing, samples were classified as good-freezability (GFE) or poor-freezability (PFE) ejaculates through cluster analyses. The SP activity levels of enzymatic (SOD and PON1) and non-enzymatic antioxidants (CUPRAC, FRAP, and TEAC) were higher (*p* < 0.05) in GFE than in PFE, whereas SP-OSI was higher (*p* < 0.05) in PFE than in GFE. In addition, the activity levels of SOD, PON1, GPX, CUPRAC, FRAP, and TEAC were positively (*p* < 0.05) related to post-thaw sperm motility and plasma membrane integrity and negatively to intracellular ROS levels. The SP-OSI was negatively correlated (*p* < 0.05) to post-thaw sperm quality parameters and positively to intracellular ROS levels. It can thus be concluded that donkey SP antioxidants are related to sperm cryotolerance and that measurements of antioxidants PON1, SOD, CUPRAC, FRAP, and TEAC, as well as SP-OSI, could be used as markers of sperm cryotolerance. Further research addressing the relationship of these antioxidants and SP-OSI with sperm cryotolerance and their potential use as freezing markers is warranted.

## 1. Introduction

In the last few decades, the donkey (*Equus asinus*) has been rediscovered in an attempt to protect biodiversity, endangered breeds, and develop marginal agricultural areas [1,2,3]. This fact, together with a growing demand for new uses (milk production, cosmetics, forestry, rural tourism, leisure...) has promoted a growing research interest in studying the reproductive characteristics of this species [3,4,5]. In this regard, the improvement of breeding management in donkeys with the use of assisted reproductive technologies, such as artificial insemination (AI) and semen cryopreservation, is crucial for their genetic selection and conservation [6].

Despite advances in cryopreservation, the use of frozen-thawed semen has been limited in donkeys due to the low conception rates obtained when jennies (0 to 36%) are inseminated compared to mares (33% to 56%) [7,8,9,10], which is attributed to the intense endometrial inflammatory response that occurs when the jenny is inseminated with frozen-thawed sperm [11,12,13]. In vivo and in vitro experiences have shown that SP, which is removed during the semen-freezing process because of its harmful effects on equids’ sperm [14], may play a key role in modulating this response [12].

Cryopreservation reduces the survival and fertilizing capacity of mammalian sperm [14]. This can be explained by the impact of freeze-thawing on plasma membrane integrity, impairing sperm motility, viability, and morphology [15,16]. Exposure of sperm to a stressful environment during cryopreservation is associated with an overproduction of reactive oxygen species (ROS) [14]. This situation could cause an imbalance in relation to the antioxidant defense, leading to oxidative stress (OS) and subsequent lipid peroxidation (LPO) of the plasma membrane [17,18,19,20]. In the case of donkeys, although it also occurs in other species, their sperm are especially sensitive to these events due to the large amount of polyunsaturated fatty acids in their plasma membrane [6,17,19,20,21]. In addition, the low amount of antioxidant enzymes within the sperm cell, together with the large number of mitochondria, makes the antioxidants (enzymatic and non-enzymatic) present in SP the main defense against the OS-induced damage [6,20,22,23]. The removal of SP before freezing, therefore, may increase sperm susceptibility to OS [6]. In recent years, many efforts have been focused on improving cryopreservation procedures in different species, analyzing the supplementation of freezing/thawing media with different antioxidants and obtaining inconsistent results [24,25,26,27,28].

In donkeys, as in other mammalian species, a high inter- and intra-male variability in the ability of sperm to withstand cryopreservation has been reported [29,30,31,32,33,34], leading to the classification of males/ejaculates as of “good” (GFE) or “poor” (PFE) freezability [17,31,35,36]. Differences in the composition of SP surrounding sperm among individuals and ejaculates could explain such differences [37,38]. In this regard, a recent study from our research group investigated whether the activities of four enzymes with antioxidant properties present in donkey SP (superoxide dismutase (SOD), catalase-like (CAT), glutathione peroxidase-like (GPX), and glutathione reductase (GSR)) were related to the sperm resilience to freezing and thawing. The results of this study show that the activity of CAT and GSR and the total and specific activity of SOD in SP were associated with donkey sperm cryotolerance [6]. As far as we are aware, however, it has not yet been reported whether other components of SP with antioxidant properties, beyond these enzymes, are related to sperm freezability in this species, nor has the activity of the previously mentioned enzymes been linked to sperm quality parameters measured by flow cytometry. Related to this, a recent investigation performed in pig SP revealed that the activity of some enzymes, such as paraoxonase (PON1), and the total non-enzymatic antioxidant capacity were positively involved in sperm cryotolerance, minimizing the OS as result of freezing and thawing [39].

Against this background, this study aimed to determine whether antioxidant components of donkey SP, including enzymes (SOD, CAT, GPX, and PON1), non-enzymatic antioxidant capacity (measured in terms of total thiol, copper-reducing antioxidant capacity (CUPRAC), ferric-reducing ability of plasma (FRAP), and Trolox equivalent antioxidant capacity (TEAC), which are assays that evaluate the total non-enzymatic antioxidant capacity of SP), and the oxidative stress index (OSI), are related to donkey sperm cryotolerance. 

## 2. Materials and Methods

### 2.1. Animals and Ejaculates

This study included 15 ejaculates from Catalonian jackasses (aged 4–11 years old) of proven fertility. All animals used were in good health condition and were housed at the Equine Reproduction Service, Autonomous University of Barcelona (Bellaterra, Cerdanyola del Vallès, Spain), a European Union-approved equine semen collection center (authorization number: ES09RS01E). This center works under strict protocols of health control and animal welfare. Because this Service already works under the approval of the Generalitat de Catalunya (Spain) and given that no manipulation on the animals beyond the collection of semen was conducted, the Ethics Committee of our institution indicated that no further ethical approval was required to carry out this study.

All animals were kept in individual paddocks and were fed a standard diet composed of mixed hay and basic concentrate. No antioxidant supplementation was used, and water was available ad libitum. A Hannover artificial vagina was used to collect the ejaculates (Minitüb GmbH, Tie-fenbach, Germany), with a nylon mesh filter connected in- line to remove the gel fraction. After removing the gel fraction, the total semen volume was evaluated, and an aliquot was used for sperm concentration assessment (Neubauer chamber, Paul Marienfeld GmbH and Co. KG; Lau-da-Königshofen, Germany). Then, the total volume of each ejaculate was divided into two equal aliquots. One was used to obtain the SP (see Section 2.2), and the other one was diluted in previously warmed (38 °C) Kenney extender (1:5v:v) [40]. This latter aliquot was utilized to evaluate sperm motility (by computer-assisted semen analysis, CASA; see Section 2.5), morphology, and viability (assessed by eosin-nigrosine staining [41]) immediately after ejaculate collection. All ejaculates fulfilled standard semen quality thresholds (>60% live sperm and >70% morphologically normal sperm). 

### 2.2. Isolation of Seminal Plasma (SP)

After collection, semen was immediately centrifuged five times at 1500× *g* for 10 min (JP Selecta S.A., Barcelona, Spain) at 4 °C. Once each centrifugation cycle finished, the supernatant was examined to ensure the absence of spermatozoa under a phase-contrast microscope (Olympus Europa, Hamburg, Germany). Five mL-aliquots of SP were subsequently stored at −80 °C. Before antioxidants measurements, SP samples were thawed on ice.

### 2.3. Measurement of Antioxidants Activity Levels and OSI in SP

The total non-enzymatic antioxidant capacity of SP was assessed in terms of CUPRAC, FRAP, and TEAC, following the protocols described by Li et al. [39], and total thiol following the procedure described by Jocelyn [42] and da Costa et al. [43]. All these procedures were previously adapted to donkey SP. The TEAC method is based on a color change by 2,2′-azinobis-3-ethylbenzothiazoline-6-sulfonate [44], the FRAP method is based on the reduction of Fe^3+^ to Fe^2+^ [45], and the CUPRAC method is based on the reduction of Cu^2+^ to Cu^1+^ [46]. The analysis of total thiol is based on the fact that thiols interact with 5, 5′-dithiobis- (2-nitrobenzoic acid) (DTNB), forming a highly colored anion with a maximum peak at 412 nm (e412 = 13,600 M^−1^ cm^−1^) [43]. All these assays were performed using an Olympus AU400 automatic chemistry analyzer (Olympus Europe GmbH, Hamburg, Germany). The results of TEAC, FRAP, and CUPRAC are expressed in mmol/L, and total thiol results are expressed in µmol/L.

Enzymatic SP antioxidants were measured as the activity of CAT, GPX, SOD, and PON1. Activities of CAT, GPX, and SOD were measured using commercially available assays following the manufacturer’s instructions (CAT: Sigma-Aldrich, St. Louis, MO, USA; GPX and SOD: Randox, Crumlin, UK). Activity of PON1 was assessed by measuring the hydrolysis of p-nitrophenyl acetate into p-nitrophenol, based on the protocol of Barranco et al. [47] adapted to donkey SP. All measurements, except for the CAT activity, were performed using an Olympus AU400 automatic chemistry analyzer. Activity of CAT was measured using a micro-plate reader (PowerWave XS; Bio-Tek Instruments). Activities of SOD and CAT were expressed as IU/mL, whereas those of PON1 and GPX were expressed as IU/L.

To calculate SP-OSI, the total oxidative status (TOS) of SP was previously measured using the method described by Erel [48] and adapted to donkey SP. This assay is based on the oxidation of ferrous ion to ferric ion in the presence of various oxidant species in acidic medium and the measurement of the ferric ion by xylenol orange. This test was also performed using an Olympus AU400 automatic chemistry analyzer. The results of TOS are expressed in μmol H_2_O_2_ equiv./L. Thereafter, SP-OSI value was calculated utilizing the following formula, as described by Wu et al. [49]: OSI (arbitrary unit) = TOS (μmol H_2_O_2_ equiv./L)/TEAC (mmol Trolox equiv./L).

Measurements of all analytes were performed in duplicate for each SP sample. In all the analytes, each test showed intra- and inter-assay coefficient variations below 10%.

### 2.4. Sperm Cryopreservation

Sperm cryopreservation was performed as previously described by Flores et al. [50]. For this purpose, before sperm cryopreservation, diluted semen was centrifuged at 600× *g* for 15 min at 20 °C (Medifriger BL-S, JP Selecta S.A., Barcelona, Spain). After the supernatant removal, the pellets were resuspended in a commercial freezing extender (Botucrio^®^, Botupharma Animal Biotechnology; Botucatu, Brazil), which contains permeable cryoprotectants (4% methylformamide and 1% glycerol). Subsequently, sperm concentration, motility, and plasma membrane integrity were reevaluated, and freezing medium (Botucrio^®^) was added until reaching a final concentration of 200 × 10^6^ viable sperm per mL (standardized in all cases). Prior to their cryopreservation using a controlled-rate freezer (Ice-Cube 14S; Minitüb), samples were packaged into 0.5-mL straws. Three stages of cooling/freezing were performed: (i) from 20 °C to 5 °C for 60 min, at a rate of −0.25 °C/min; (ii) from 5 °C to −90 °C for 20 min, at a rate of −4.75 °C/min; and (iii) from −90 °C to −120 °C for 2.7 min, at a rate of −11.11 °C/min. Once the last freezing step finished, straws were immediately submerged into liquid nitrogen and kept in tanks until their thawing and analysis. The thawing protocol consisted of incubating the straws for 30 s in a water bath at 38 °C followed by dilution with three volumes of Kenney extender, also prewarmed at 38°C (final concentration: 50 × 10^6^ sperm/mL)

### 2.5. Assessment of Sperm Motility

Before and after cryopreservation, sperm motility was evaluated using a CASA system (Proiser S.L.; Valencia, Spain). For that, 5 μL of each semen sample (diluted in Kenney extender at a concentration of 50 × 10^6^ spermatozoa/mL) was placed onto a previously heated (38 °C) Makler chamber (Sefi Medical Instruments; Haifa, Israel). Then, samples were assessed under a 10× negative phase-contrast objective using an Olympus B×41 microscope (Olympus, Tokyo, Japan) that also had a plate heated to 38 °C. A minimum of 1000 sperm cells per analysis were counted. Percentages of total (TM, %) and progressive motility (PM, %) were recorded in each evaluation together with kinetic parameters as follows: straight-line velocity (VSL, μm/s), which is the mean path velocity of the sperm head along a straight line from its first to its last position; curvilinear velocity (VCL, μm/s), which is the mean path velocity of the sperm head along its actual trajectory; average path velocity (VAP, μm/s), which is the mean velocity of the sperm head along its average trajectory; percentage of straightness (STR, %), which is the quotient between VSL and VAP multiplied by 100; percentage of linearity (LIN, %), which is the quotient between VSL and VCL multiplied by 100; percentage of oscillation (WOB, %), which is the quotient between VAP and VCL multiplied by 100; frequency of head displacement (BCF, Hz), which is the frequency at which the head crosses the actual sperm track; and mean amplitude of lateral head displacement (ALH, μm), which is the mean value of the extreme side-to-side movement of the sperm head in each beat cycle. The settings of CASA used were those recommended by the provider, i.e., frames/s: 25 images captured per second; connectivity: 6; particle area >4 and <75 μm^2^; minimum number of images to calculate the ALH: 10. Cut-off value for motile spermatozoa was VAP ≥ 10 μm/s and for progressively motile spermatozoa was STR ≥ 75%.

### 2.6. Flow Cytometry Analyses

Flow cytometry was used to determine the integrity and lipid disorder of sperm plasma membrane, mitochondrial membrane potential (MMP), and intracellular levels of overall ROS, superoxides, and calcium in post-thawed semen samples, following the recommendations set by the International Society for Advancement of Cytometry [51].

In this study, the Cell Lab Quanta SC™ flow cytometer was used (Beckman Coulter, Fullerton, CA, USA), and particles were excited with an argon laser (488 nm) at a power of 22 mW. Firstly, sperm concentration was adjusted to 1 × 10^6^ sperm/mL. Before starting to use the cytometer, the electronic volume (EV) channel was calibrated with 10-μm-diameter fluorescent beads (Beckman Coulter) following the provider’s instructions. The analyzer threshold was established to exclude cell aggregates (particles with a diameter >12 μm) and cell debris (particles with a diameter <7 μm), and the flow rate was set at 4.17 μL/min. Based on EV and side scatter (SS) distributions, sperm cells were gated. Three different optical filters were used (FL1 for the analysis of H_2_DCFDA, SYBR14, YO-PRO-1, JC1 monomers, and Fluo3, detection width: 505–545 nm; FL2 for the analysis of JC1 aggregates, detection width: 560–590 nm; FL3 for the analysis of PI, HE, and M540, detection width: 655–685 nm). Compensation was used when required to minimize fluorescence spill-over into a different channel. All events information was collected in List-mode Data files (EV, SS, FL1, FL2, and FL3) and processed using the Cell Lab Quanta SC MPL Analysis Software (version 1.0; Beckman Coulter). Data were corrected in all assessments based on the percentage of debris particles (SYBR14^–^/propidium iodide (PI)^–^) determined through SYBR14/PI staining, as previously described by Petrunkina et al. [52],. All fluorochromes used were purchased from Molecular Probes^®^ (Invitrogen^®^, Thermo Fisher Scientific, Waltham, MA, United States) and diluted in dimethyl sulfoxide (DMSO).

#### 2.6.1. Evaluation of Sperm Plasma Membrane Integrity

Sperm plasma membrane integrity was assessed using the LIVE/DEAD Sperm Viability Kit (SYBR14, viable spermatozoa, and PI, non-viable spermatozoa), following a protocol modified for donkey sperm, adapted from Garner and Johnson [53]. Briefly, sperm samples were incubated first with SYBR14 (final concentration: 100 nM) for 10 min at 38 °C and later with PI (final concentration: 12 mM) for 5 min at 38 °C. All incubations were performed protecting samples from the light. To measure the green fluorescence from SYBR14, FL1 was used, and to detect the red fluorescence from PI, FL3 was used. Three sperm populations were identified: (1) sperm with an intact plasma membrane (SYBR14^+^/PI^−^; viable sperm); (2) sperm with a damaged plasma membrane (SYBR14^−^/PI^+^); and (3) sperm with a damaged plasma membrane (SYBR14^+^/PI^+^). Non-sperm debris particles were identified as those that were not stained with either SYBR14 or PI (SYBR14^−^/PI^−^). SYBR14 spill-over into the PI channel was compensated (2.45%).

#### 2.6.2. Evaluation of Membrane Lipid Disorder

Membrane lipid disorder was determined through staining with merocyanine 540 (M540) and YO-PRO-1 fluorochromes. Sperm were incubated with M540 (final concentration: 2.6 mM) and YO-PRO-1 (used as a vital stain; final concentration: 25 nM) at 38 °C for 10 min in the dark [54]. Four sperm populations were detected: (1) viable sperm with low membrane lipid disorder (M540^−^/YO-PRO-1^−^); (2) viable sperm with high membrane lipid disorder (M540^+^/YO-PRO-1^−^); (3) non-viable sperm with low membrane lipid disorder (M540^−^/YO-PRO-1^+^); and (4) non-viable sperm with high membrane lipid disorder (M540^+^/YO-PRO-1^+^). Percentages of debris particles found in SYBR14/PI staining (SYBR14^−^/PI^−^) were subtracted from those of viable sperm with low membrane lipid disorder (M540^−^/YO-PRO-1^−^); thereafter, the percentages of all sperm populations were recalculated.

#### 2.6.3. Evaluation of Mitochondrial Membrane Potential

The MMP was determined using JC-1 (5,50,6,60-tetrachloro-1,10,3,3′tetraethyl-benzimidazolylcarbocyanine iodide). Under dark conditions, sperm samples were stained with JC-1 (final concentration: 0.5 mM) at 38 °C for 30 min. When mitochondrial membrane potential is low, JC-1 monomers emit green fluorescence (JC-1_mon_), which is collected through FL1. If mitochondrial membrane potential is high, JC-1 forms aggregates emitting orange fluorescence (JC-1_agg_), which is detected through FL2. Three sperm populations were distinguished: (1) green-stained sperm with low mitochondrial membrane potential (low MMP); (2) orange-stained sperm (high MMP); and (3) sperm with heterogeneous mitochondria stained both green and orange in the same cell (intermediate MMP). Spill-over of FL1 into FL2 channel was compensated (68.50%). Percentages of debris particles found in SYBR14/PI staining (SYBR14^−^/PI^−^) were subtracted from those of sperm with low MMP, and percentages of all sperm populations were recalculated.

#### 2.6.4. Evaluation of Intracellular ROS Levels

Intracellular ROS levels were determined through two oxidation-sensitive fluorescent probes: hydroethidine (HE) and 2,7-dichlorodihydrofluorescein and diacetate (H_2_DCFDA), which detect superoxide anion (∙O_2_^−^) and overall ROS, respectively [55]. Following a modified procedure from Guthrie and Welch [56], a simultaneous differentiation of viable and non-viable sperm was performed using YO-PRO-1 (HE) or PI (H_2_DCFDA).

For ∙O_2_^−^, sperm samples were mixed with Hydroethidine (HE, final concentration: 4 mM) and YO-PRO-1 (final concentration: 25 nM) and then incubated at 25 °C for 30 min in the dark. Oxidation of HE to ethidium (E^+^) was detected through FL3 (red fluorescence), and green fluorescence from YO-PRO-1 was collected through FL1. Four sperm populations were identified: (1) non-viable sperm with low ∙O_2_^−^ levels (E^−^/YO-PRO-1^+^); (2) non-viable sperm with high ∙O_2_^−^ levels (E^+^/YO-PRO-1^+^); (3) viable sperm with low ∙O_2_^−^ levels (E^−^/YO-PRO-1^−^); and (4) viable sperm with high ∙O_2_^−^ levels (E^+^/YO-PRO-1^−^). YO-PRO-1 spill-over into the FL3 channel was compensated (5.06%).

For overall ROS, sperm samples were stained with H_2_DCFDA (final concentration: 140 mM) and PI (final concentration: 12 mM) and incubated at 25 °C for 30 min in the dark. Oxidation of H_2_DCFDA to dichlorofluorescein (DCF^+^) was detected through FL1 (green fluorescence), and red fluorescence from PI was collected through FL3. Four sperm populations were identified: (1) viable sperm with high ROS levels (DCF^+^/PI^−^); (2) non-viable sperm with high ROS levels (DCF^+^/PI^+^); (3) viable sperm with low ROS levels (DCF^−^/PI^−^); and (4) non-viable sperm with low ROS levels (DCF^−^/PI^+^). DCF^+^ spill-over into the FL3 channel was compensated (2.45%).

#### 2.6.5. Evaluation of Intracellular Calcium Levels

Previous studies in pig sperm found that Fluo3-acetomethoxyester fluorochrome (Fluo3) mainly stains mitochondrial calcium [57]. For this reason, we combined this fluorochrome with PI (Fluo3/PI), following the method described by Kadirvel et al. [58]. Four sperm populations were identified: (1) viable sperm with low levels of intracellular calcium (Fluo3^−^/PI^−^); (2) viable sperm with high levels of intracellular calcium (Fluo3^+^/PI^−^); (3) non-viable sperm with low levels of intracellular calcium (Fluo3^−^/PI^+^); and (4) non-viable sperm with high levels of intracellular calcium (Fluo3^+^/PI^+^). The FL3 overflow in the FL1 channel (28.72%) and the FL1 overflow in the FL3 channel (2.45%) were compensated.

### 2.7. Experimental Design

A total of 15 ejaculates from 15 different Catalan donkeys were used. After collection, each ejaculate was divided into two aliquots. The first aliquot was centrifuged (see Section 2.2) to obtain SP samples for the measurement of the activity levels of enzymatic and non-enzymatic antioxidants and OSI. The second aliquot was cryopreserved following the previously described procedure (see Section 2.4). Before cryopreservation, sperm concentration, motility, morphology, and viability were evaluated. Ten minutes after thawing, sperm motility, plasma membrane integrity and lipid disorder, MMP, and intracellular levels of overall ROS, superoxides and calcium were evaluated in each semen sample. Based on the percentage of total motile sperm (TM) and the percentage of viable sperm (SYBR14^+^/PI^−^) after thawing, donkey ejaculates were classified as GFE and PFE by hierarchical clustering.

### 2.8. Statistical Analysis

Data analyses were performed using the R statistical package (V 4.0.3, R Core Team; Vienna, Austria), and graphs were prepared with the GraphPad Prism software (V 8.4.0, GraphPad Software LLC; San Diego, CA, USA). First, Shapiro–Wilk and Levene tests were run to verify normal distribution and homogeneity of variances, respectively. If necessary, data were transformed using the square root of arcsin (arcsin √x) to keep parametric assumptions. The minimum level of statistical significance for all analyzes was set at *p* ≤ 0.05. Results are shown as mean ± standard error of the mean (SEM).

#### 2.8.1. Hierarchical Clusters and Comparison between GFE and PFE

Classification of the 15 ejaculates into groups of GFE and PFE was carried out by means of a hierarchical cluster analysis of complete linkage using the Euclidean distances from the percentages of TM and viable sperm (SYBR14^+^/PI^−^) obtained in each sample after thawing, as described in Morató et al. [59].

The SP activity levels of enzymatic (PON1, CAT, GPX, and SOD) and non-enzymatic antioxidants (total thiol, CUPRAC, FRAP, and TEAC), as well as SP-OSI in donkey ejaculates were compared between GFE and PFE by means of a *t*-test for independent samples. When, even after being linearly transformed, these data did not match with parametric assumptions, the Mann–Whitney test was used as an alternative. 

#### 2.8.2. Correlation Matrix

Pearson’s coefficients were calculated to develop the correlation matrix between the SP activity levels of enzymatic (PON1, CAT, GPX, and SOD) and non-enzymatic antioxidants (total thiol, CUPRAC, FRAP, and TEAC), as well as SP-OSI values in donkey ejaculates, and TM, PM, kinematic parameters (VCL, VSL, VAP, LIN, STR, WOB, ALH, and BCF), plasma membrane integrity (SYBR14^+^/PI^−^), MMP (JC-1_agg_), intracellular ROS levels (DCF^+^/PI^−^), intracellular superoxides levels (E^+^/YO-PRO-1^−^), intracellular calcium levels (Fluo3^+^/PI^−^), and plasma membrane lipid disorder (M540^+^/YO-PRO-1^−^), obtained in each sample after thawing.

## 3. Results

### 3.1. Classification of Donkey Ejaculates into GFE and PFE Groups According to Their Post-Thaw Sperm Quality and Functionality Parameters

**Figure 1:**
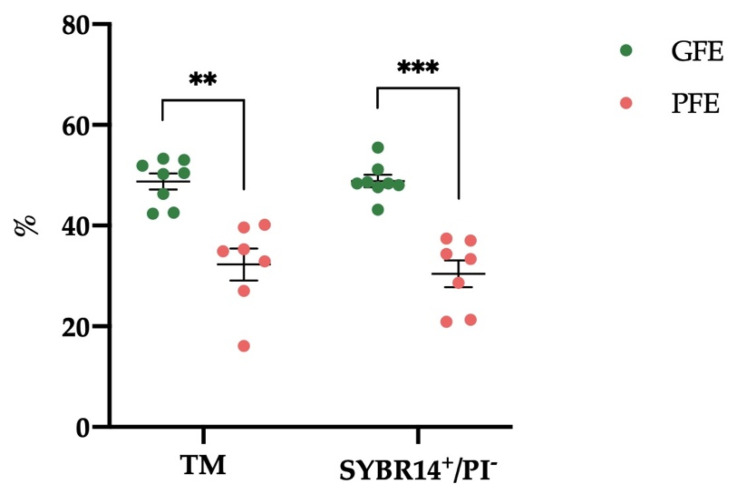
Mean ± SEM of the percentages of total sperm motility (TM) and of sperm with an intact plasma membrane (SYBR14^+^/PI^−^, viable sperm) after thawing in donkey ejaculates (*n* = 15) classified as good-freezability (GFE) or poor-freezability ejaculates (PFE). The dots represent the TM and SYBR14^+^/PI^−^ individual values in GFE and PFE. ** *p* ≤ 0.01; *** *p* ≤ 0.001.

According to TM and sperm viability (SYBR14^+^/PI^−^) obtained after thawing, the 15 ejaculates were classified (by hierarchical clustering, *p* < 0.01) into GFE (*n* = 8) and PFE (*n* = 7). The percentages of TM and viable (SYBR14^+^/PI^−^) sperm after thawing were significantly higher (*p* < 0.01 and *p* < 0.001, respectively; Figure 1) in GFE (48.76 ± 1.58 and 48.85 ± 1.23%, respectively) than in PFE (32.29 ± 3.17 and 30.43 ± 2.64%, respectively).

The ejaculates classified as GFE presented a range of TM between 42.35 and 53.27% and a range of SYBR14^+^/PI^−^ between 43.16 and 55.48%, whereas the PFE were in ranges of TM and SYBR14^+^/PI^−^ between 16.09 and 40.14% and between 20.89 and 37.40%, respectively (Tables S1 and S2).

### 3.2. Activity Levels of Enzymatic Antioxidants in Donkey SP

**Figure 2:**
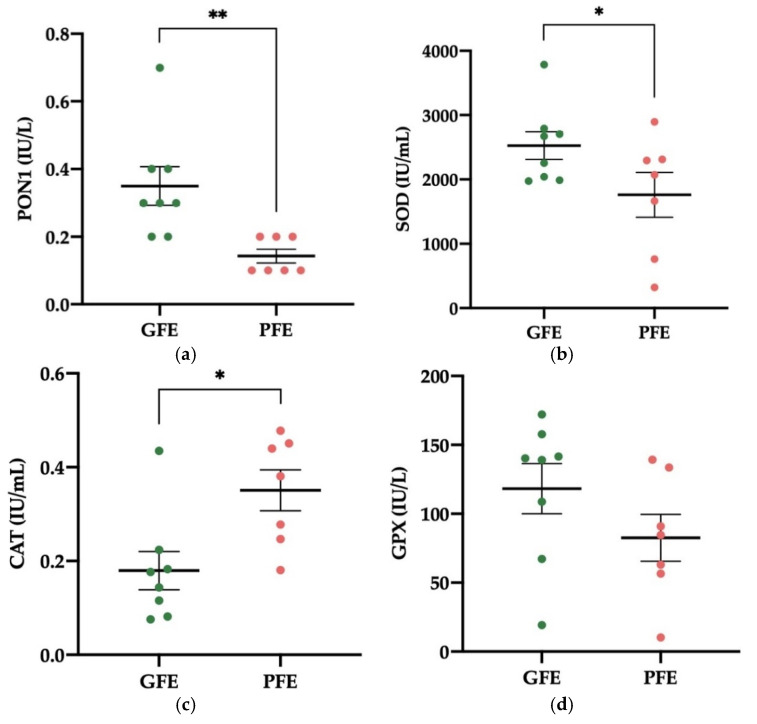
Mean ± SEM of the activity levels of paraoxonase type 1 (PON1, **a**), superoxide dismutase (SOD, **b**), catalase-like (CAT, **c**), and glutathione peroxidase-like (GPX, **d**) in seminal plasma (SP) in donkey ejaculates classified as having good (GFE, *n* = 8) or poor freezability (PFE, *n* = 7). * *p* ≤ 0.05; ** *p* ≤ 0.01.

The activity levels of PON1 and SOD in SP were significantly higher in GFE (*p* < 0.01 and *p* < 0.05, respectively; 0.35 ± 0.06 and 2527 ± 216 IU/mL, respectively; Figure 2a,b) than in PFE (0.14 ± 0.02 and 1760 ± 347 IU/mL, respectively). On the contrary, the SP of PFE showed higher activity levels of CAT than that of GFE (0.35 ± 0.04 vs. 0.18 ± 0.04 IU/mL, respectively; *p* < 0.05; Figure 2c). Moreover, the activity levels of GPX in SP did not differ significantly between GFE and PFE (Figure 2d). Table S3 shows the mean ± SEM and range of each enzymatic antioxidant’s activity levels in SP samples of all donkey ejaculates included in the study.

### 3.3. Activity Levels of Non-Enzymatic Antioxidants in Donkey SP

**Figure 3:**
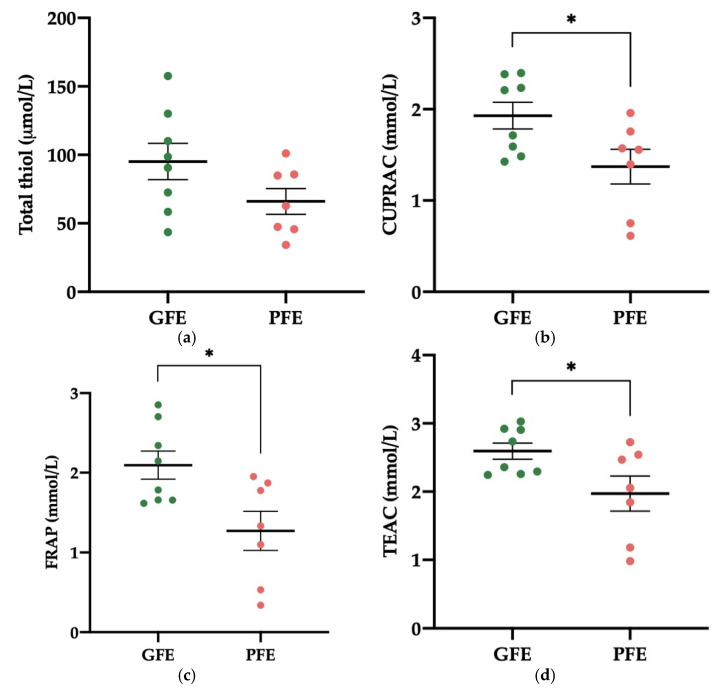
Mean ± SEM of the activity levels of non-enzymatic antioxidants (measured in terms of total thiol (**a**), cupric reducing antioxidant capacity (CUPRAC, **b**), plasma iron-reducing capacity (FRAP, **c**), and Trolox equivalent antioxidant capacity (TEAC, **d**)) in seminal plasma (SP) of donkey ejaculates classified as having good (GFE, *n* = 8) or poor freezability (PFE, *n* = 7). * *p* ≤ 0.05.

No differences were found in the levels of total thiol between GFE and PFE (Figure 3a). The activity levels of CUPRAC, FRAP, and TEAC in SP, however, were higher (*p* < 0.05) in donkey ejaculates classified as GFE than in those classified as PFE (Figure 3b–d). The CUPRAC values were 1.93 ± 0.15 and 1.37 ± 0.19 mmol/L, respectively. The FRAP values were 2.09 ± 0.18 and 1.27 ± 0.25 mmol/L, respectively. Meanwhile, TEAC values were 2.59 ± 0.12 and 1.97 ± 0.26 mmol/L, respectively. Table S3 shows the mean ± SEM and range of each non-enzymatic antioxidant’s activity levels in SP samples of all donkey ejaculates included in the study.

### 3.4. Levels of OSI in Donkey SP

**Figure 4:**
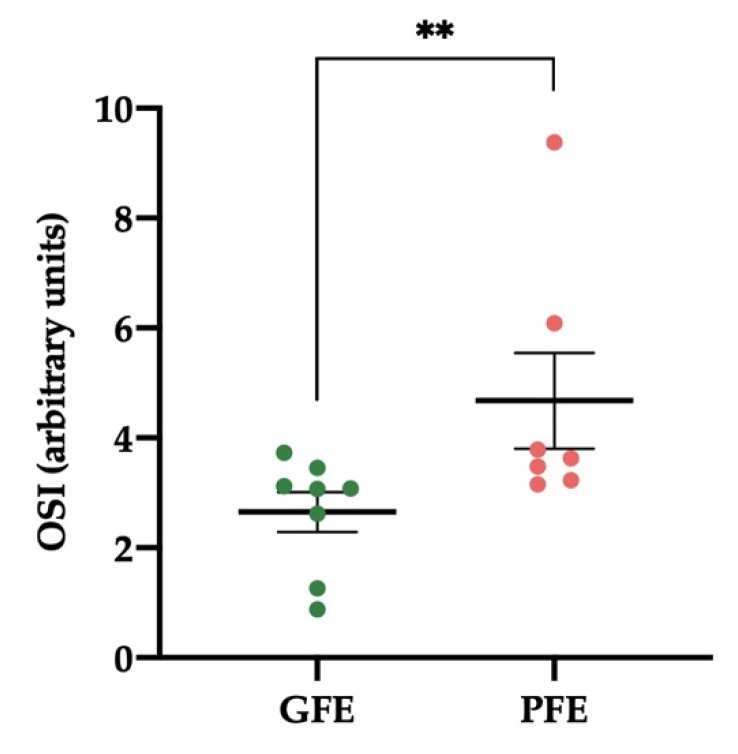
Mean ± SEM of the levels of seminal oxidative stress index (OSI) of donkey ejaculates classified as having good (GFE, *n* = 8) or poor freezability (PFE, *n* = 7). ** *p* ≤ 0.01.

The levels of OSI in SP from donkey ejaculates were significantly lower (*p* < 0.01) in GFE than in PFE (Figure 4). The values were 2.65 ± 0.36 and 4.68 ± 0.87, respectively. TOS levels were 6.85 ± 0.96 and 8.04 ± 0.34 µmol/L in GFE and PFE, respectively. Table S3 shows the mean ± SEM and range of OSI and TOS in SP of all donkey ejaculates included in the study.

### 3.5. Correlations of the Activity Levels of Enzymatic and Non-Enzymatic Antioxidants and OSI in Donkey SP with Post-Thaw Sperm Motility Parameters

**Figure 5:**
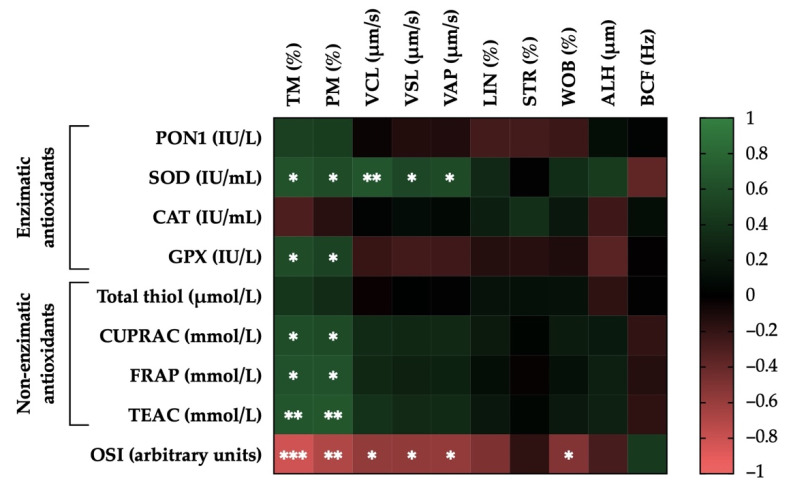
Correlations of the activity levels of enzymatic (paraoxonase type 1, PON1; superoxide dismutase, SOD; catalase-like, CAT; and glutathione peroxidase-like, GPX) and non-enzymatic antioxidants (measured in terms of total thiol; cupric-reducing antioxidant capacity, CUPRAC; plasma iron-reducing capacity, FRAP; and Trolox equivalent antioxidant capacity, TEAC) and oxidative stress index (OSI) in donkey seminal plasma (SP; *n* = 15) with post-thaw sperm motility parameters (percentage of total motility, TM; percentage of progressively motile spermatozoa, PM; curvilinear velocity, VCL; straight-line velocity, VSL; average path velocity, VAP; linearity coefficient, LIN; straightness coefficient, STR; wobble coefficient, WOB; amplitude of lateral head displacement, ALH; beat-cross frequency, BCF). The scale colors (1 to −1) indicate whether the correlation is positive (green) or negative (red). * *p* ≤ 0.05; ** *p* ≤ 0.01; *** *p* ≤ 0.001.

Figure 5 shows the correlation matrix between sperm motility and SP antioxidant parameters. Significant correlations between the activity levels of enzymatic and non-enzymatic antioxidants and OSI in SP and post-thaw sperm motility parameters were observed. For example, the activity levels of SOD and GPX were positively correlated with TM (r = 0.64, *p* < 0.05 and r = 0.59, *p* < 0.05, respectively) and with PM (r = 0.59, *p* < 0.05 and r = 0.52, *p* < 0.05, respectively). Furthermore, the activity levels of SOD were positively correlated with kinematic parameters (r = 0.66, *p* < 0.01 for VCL; r = 0.55, *p* < 0.05 for VSL; and r = 0.57, *p* < 0.05 for VAP). Similarly, the activity levels of non-enzymatic antioxidants (measured in terms of CUPRAC, FRAC, and TEAC) were positively correlated with TM (r = 0.60, *p* < 0.05; r = 0.64, *p* < 0.05; and r = 0.65, *p* < 0.01, respectively) and with PM (r = 0.58, *p* < 0.05; r = 0.63, *p* < 0.05; and r = 0.66, *p* < 0.01, respectively).

On the contrary, the levels of SP-OSI were negatively correlated with TM (r = −0.82, *p* < 0.001), PM (r = −0.70, *p* < 0.01), and the following kinematic parameters: VCL (r = −0.58, *p* < 0.05), VSL (r = −0.57, *p* < 0.05), VAP (r = −0.59, *p* < 0.05), and WOB (r = −0.52, *p* < 0.05). Table S1 shows the mean ± SEM and range of each sperm motility parameter after thawing in GFE and PFE.

### 3.6. Correlations of the Activity Levels of Enzymatic and Non-Enzymatic Antioxidants and OSI in Donkey SP with Post-Thaw Sperm Functionality Parameters

**Figure 6:**
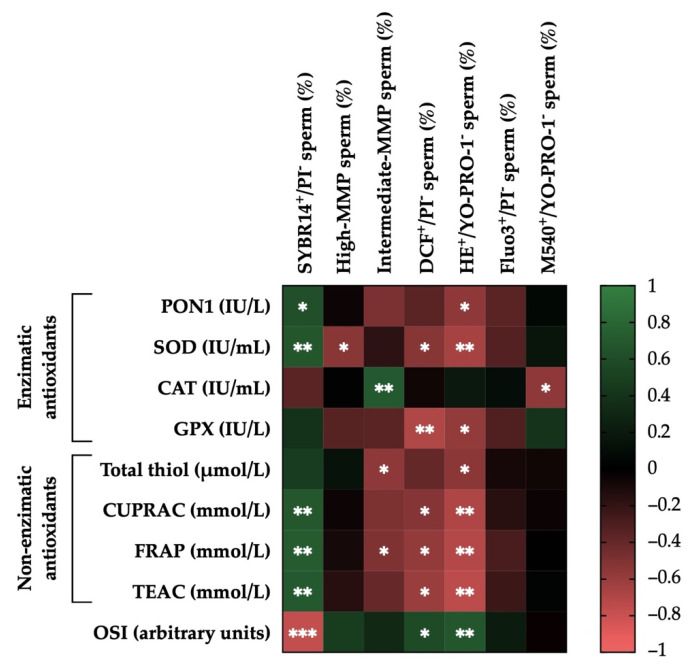
Correlations of the activity levels of enzymatic (paraoxonase type 1, PON1; superoxide dismutase, SOD; catalase-like, CAT; and glutathione peroxidase-like, GPX) and non-enzymatic antioxidants (measured in terms of total thiol; cupric reducing antioxidant capacity, CUPRAC; plasma iron-reducing capacity, FRAP; and Trolox equivalent antioxidant capacity, TEAC) and oxidative stress index (OSI) in donkey seminal plasma (SP; *n* = 15) with post-thaw sperm functionality parameters (plasma membrane integrity, SYBR14^+^/PI^−^; mitochondrial membrane potential, MMP; intracellular ROS levels, DCF^+^/PI^−^; intracellular superoxide levels, E^+^/YO-PRO-1^−^; intracellular calcium levels, Fluo3^+^/PI^−^; plasma membrane lipid disorder, M540^+^/YO-PRO-1^−^). The scale colors (1 to −1) indicate whether the correlation is positive (green) or negative (red). * *p* ≤ 0.05; ** *p* ≤ 0.01; *** *p* ≤ 0.001.

Figure 6 shows the correlation matrix between sperm functionality and SP antioxidant parameters. Significant correlations between the activity levels of enzymatic or non-enzymatic antioxidants and OSI in donkey SP and post-thaw sperm functionality parameters were observed. For example, activity levels of PON1 and SOD were positively correlated with plasma membrane integrity (SYBR14^+^/PI^−^; r = 0.61, *p* < 0.05; and r = 0.67, *p* < 0.01, respectively). On the contrary, activity levels of SOD and GPX were negatively correlated with intracellular ROS levels (DCF^+^/PI^−^; r = −0.53, *p* < 0.05; and r = −0.70, *p* < 0.01, respectively) and with intracellular superoxide levels (HE^+^/YO-PRO-1^−^; r = −0.66, *p* < 0.01; and r = −0.58, *p* < 0.05, respectively), whereas activity levels of PON1 were negatively correlated only with the latter (HE^+^/YO-PRO-1^−^; r = −0.57, *p* < 0.05). In addition, activity levels of SOD were negatively correlated with the proportion of sperm with high MMP (r = −0.54, *p* < 0.05). Activity levels of CAT were positively correlated with the proportion of sperm with intermediate MMP (r = 0.69, *p* < 0.01) and negatively with plasma membrane lipid disorder (M540^+^/YO-PRO-1^−^; r = −0.57, *p* < 0.05).

Regarding the activity levels of non-enzymatic antioxidants, CUPRAC, FRAP, and TEAC showed strong positive correlations with the integrity of plasma membrane (SYBR14^+^/PI^−^; r = 0.69, *p* < 0.01; r = 0.71, *p* < 0.01; and r = 0.69, *p* < 0.01, respectively). In contrast, they were negatively correlated with intracellular ROS levels (DCF^+^/PI^−^; r = −0.53, *p* < 0.05; r = −0.58, *p* < 0.05; and r = −0.61, *p* < 0.05, respectively) and strongly with intracellular superoxide levels (HE^+^/YO-PRO-1^−^; r = −0.69, *p* < 0.01; r = −0.71, *p* < 0.01; and r = −0.75, *p* < 0.01, respectively). Activity levels of total thiol were also negatively correlated with HE^+^/YO-PRO-1^−^ (r = −0.55, *p* < 0.05). Furthermore, the activity levels of total thiol and FRAC were also negatively correlated with the proportion of sperm with intermediate MMP (r = −0.57, *p* < 0.05; and r = −0.50, *p* < 0.05, respectively).

Finally, the SP-OSI showed a strong negative correlation with the integrity of plasma membrane (SYBR14^+^/PI^−^; r = −0.77, *p* < 0.001) and was positively correlated with intracellular ROS levels (DCF+/PI^−^; r = 0.57, *p* < 0.05) and intracellular superoxide levels (HE^+^/YO-PRO-1^−^; r = 0.67, *p* < 0.01). Table S2 shows the mean ± SEM and range of each sperm function parameter after thawing in GFE and PFE.

### 3.7. Correlations between the Activity Levels of Enzymatic and Non-Enzymatic Antioxidants and OSI in Donkey SP

**Figure 7:**
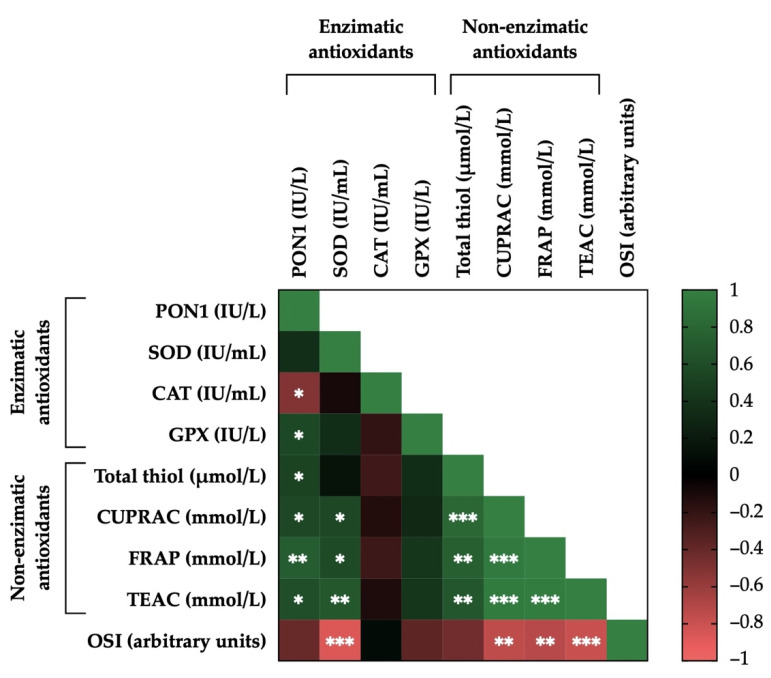
Correlations between the activity levels of enzymatic (paraoxonase type 1, PON1; superoxide dismutase, SOD; catalase-like, CAT; and glutathione peroxidase-like, GPX) and non-enzymatic antioxidants (measured in terms of total thiol; cupric reducing antioxidant capacity, CUPRAC; plasma iron-reducing capacity, FRAP; and Trolox equivalent antioxidant capacity, TEAC) and oxidative stress index (OSI) in donkey seminal plasma (SP; *n* = 15). The scale colors (1 to −1) indicate whether the correlation is positive (green) or negative (red). * *p* ≤ 0.05; ** *p* ≤ 0.01; *** *p* ≤ 0.001.

Figure 7 shows the correlation matrix and the relationship between activity levels for all enzymatic and non-enzymatic antioxidants and OSI in donkey SP. Positive correlations were observed for GPX (r = 0.54, *p* < 0.05) and the four assays used for evaluating non-enzymatic antioxidants (total thiol: r = 0.50, *p* < 0.05; CUPRAC: r = 0.56, *p* < 0.05; FRAP: r = 0.73, *p* < 0.01; and TEAC: r = 0.61, *p* < 0.05) with PON1. The activity levels of four non-enzymatic antioxidants showed strong positive correlations with each other: CUPRAC (r = 0.81, *p* < 0.001), FRAP (r = 0.75, *p* < 0.01), and TEAC (r = 0.68, *p* < 0.01) with total thiol; FRAP (r = 0.95, *p* < 0.001) and TEAC (r = 0.96, *p* < 0.001) with CUPRAC; and TEAC (r = 0.97, *p* < 0.001) with FRAP. Furthermore, the activity levels of CUPRAC, FRAP, and TEAC were positively correlated with SOD (r = 0.56, *p* < 0.05; r = 0.58, *p* < 0.05; and r = 0.68, *p* < 0.01, respectively). On the contrary, negative correlations between CAT and PON1 (r = −0.58, *p* < 0.05) were observed. Likewise, levels of SP-OSI showed strong negative correlations with SOD (r = −0.87, *p* < 0.001), CUPRAC (r = −0.75, *p* < 0.01), FRAP (r = −0.72, *p* < 0.01) and TEAC (r = −0.80, *p* < 0.001). Table S3 shows the mean ± SEM and range of each enzymatic and non-enzymatic antioxidant’s activity levels, as well as OSI in SP samples of all donkey ejaculates included in the study.

## 4. Discussion

The present study demonstrated that the activity levels of some SP components with antioxidant properties, both enzymatic and non-enzymatic, as well as the SP-OSI were related to the quality and functionality parameters (motility, plasma membrane integrity, and intracellular levels of ROS) of frozen-thawed donkey sperm. The main finding, however, was that the activity levels of enzymatic antioxidants SOD and PON1, those of non-enzymatic antioxidants (measured in terms of CUPRAC, FRAP, and TEAC), and the OSI assessed in donkey SP were directly related to the sperm resilience to cryopreservation in this species.

For a long time, SP was regarded as a passive medium that accompanies sperm during and after ejaculation [60]. More than a natural diluent and a transport vehicle, nevertheless, SP has recently been considered as an important player for fertility in mammals, both regarding sperm function and female-reproductive-tract-related processes [22,60,61]. In effect, this fluid also regulates the immune response of the female reproductive tract generated by the presence of semen [60,61,62,63]. It is well-known that SP represents the most important source of enzymatic and non-enzymatic antioxidants for sperm, being capable of eliminating the excess of ROS that induces OS in semen [6,17,22,64,65].. It is worth mentioning that the composition of SP as well as the capacity of sperm to withstand freeze-thawing differ not only between species, but also between and within individuals [6,17,63,66]. In addition, SP components, mainly those with antioxidant properties, may be related to the cryotolerance of sperm to freeze-thawing processes [6,17]. This study, therefore, aimed to evaluate whether differences between donkey ejaculates/individuals in the resilience of their sperm to cryopreservation could be explained by differences in their SP-antioxidant composition.

As far as we are aware, this is the first report addressing the relationship of the levels of antioxidants, enzymatic and non-enzymatic, and OSI in SP with the quality and functionality parameters of donkey sperm after freeze-thawing. When enzymatic antioxidants PON1 and SOD and non-enzymatic antioxidants (measured in terms of CUPRAC, FRAP, and TEAC) of SP were evaluated, their levels were higher in ejaculates categorized as GFE than in those graded as PFE. Activity levels of enzymatic antioxidant CAT and SP-OSI, however, were higher in ejaculates classified as PFE compared to those classified as GFE. These results are in agreement with those previously observed in donkey semen in the case of SOD [6] and in pig semen in the case of SOD, PON1, CAT, FRAP, and TEAC [39]. However, our results differ from those previously reported in donkey semen in the case of CAT. The lower CAT activity levels we found in donkey GFE ejaculates could be related to the high activity levels of PON1 detected in this group. PON1, therefore, might be playing a more relevant role in the control of ROS in donkey semen than CAT. This hypothesis could explain the negative correlation observed between CAT and PON1 activity levels. We also found differences in CUPRAC levels compared to pig SP, where these levels were lower in the fraction of semen with better freezability [39]. This could rely on the distinct SP composition and the different effect of antioxidants depending on species [17], reproductive season [22,67], and age [67]. Based on these considerations, it is reasonable to suggest that the resilience of donkey sperm to cryopreservation may be determined by the high activity levels of enzymatic antioxidants PON1 and SOD and total non-enzymatic antioxidants (measured in terms of CUPRAC, FRAP, and TEAC) in SP. The brief contact of sperm with SP before its removal could be sufficient to exert the beneficial effect of antioxidants on donkey sperm cryotolerance, which is similar to findings previously reported in donkey and horses [6,17].

Regarding the enzymatic antioxidants of SP analysed in this study, our results support previous studies that highlight the relevance of SOD for sperm cryotolerance in donkeys, horses, pigs, humans, and buffaloes [6,17,39,67,68], where SOD was positively related to post-thaw motility [39,67,68] and viability [6,17,39] and negatively to intracellular ROS levels [39]. This neutralization of ROS is produced through a cascade of reactions initiated by SOD, an essential antioxidant that avoids OS [39,69]. SOD protects the cell against O_2_^−^, as it catalyses the dismutation of this anion into H_2_O_2_ [70]. Furthermore, this reaction prevents the formation of the highly reactive hydroxyl radical (OH^−^) that occurs when O_2_^−^ and H_2_O_2_ react with the ferric ion [70,71]. In our study, the positive and negative correlation of SOD activity levels with post-thaw sperm quality and with ROS levels, respectively, together with the higher SOD activity levels found in GFE in comparison to PFE, would confirm that, in donkey semen, SOD is the main antioxidant SP enzyme involved in the detoxification of ROS [6]. Hence, the measurement of SOD activity levels in donkey SP could be used as a marker of sperm cryotolerance in this species. However, although the protocol used in this work and similar protocols to measure intracellular O_2_^−^ levels have been validated and tested in different studies and species [56,72,73], they could be evaluating O_2_^−^ imprecisely, as reported by Robinson et al. [74] and more recently by Gallo et al. [75]. When oxidized, hydroethidine generates two red fluorescent products, 2-hydroxyethidium (2-OH-E^+^) and ethidium (E^+^). The former selectively detects O_2_^−^, while the latter binds to DNA [74,75,76]. Therefore, the protocols used to assess intracellular O_2_^−^ levels should maximize the detection of specific product 2-OH-E^+^. This issue should be considered in future studies.

In order for SOD to fulfil its function as an antioxidant, it is combined with additional enzymes able to metabolize the H_2_O_2_ generated [77], because an excess of H_2_O_2_ is toxic to tissues and cells [78]. In this sense, the activity of SOD is completed with that of enzymes such as GPX and CAT [70,78]. Regarding GPX, which is an enzyme whose function is to reduce H_2_O_2_ to water [17,70], its levels did not differ between GFE and PFE, which is in agreement with the results reported in previous studies using frozen-thawed donkey and horse sperm [6,17]. Curiously, our results show a positive relationship between the activity levels of GPX in SP and both TM and PM, differing from previous studies conducted in fresh and frozen-thawed donkey semen [6,22]. We also observed a negative correlation between GPX activity in SP and intracellular ROS levels, a parameter that was not evaluated in the aforementioned studies carried out in donkeys [6,22]. At this point, it is important to mention that previous reports also found differences in the functional role of GPX between species; in fresh, liquid-stored, or frozen semen; as well as between studies conducted in the same species [6,17]. In effect, while kinetic sperm parameters have been reported to be negatively correlated to GPX activity in sperm and SP from rams [79], this activity has been found to be positively related to the quality parameters and fertilizing capacity of fresh sperm from Arabian stallions. Other studies carried out in horses, however, reported a negative relationship between GPX activity in SP and motility of fresh and frozen-thawed sperm [17,22]. Taking into consideration our results and the previous studies in frozen-thawed donkey sperm [6], one could posit that the lack of differences in GPX activity between GFE and PFE would indicate that this enzyme cannot be used as a marker of sperm cryotolerance in donkeys.

The importance of CAT, an enzyme whose function is to break down H_2_O_2_ into water and molecular oxygen (O_2_) and thus functions as a ROS scavenger, has been previously described in mammalian sperm [6,70,80]. In fact, CAT has been shown to counteract the deleterious effects of OS on human [81,82], mouse [83], horse [84], and pig sperm [56], which is mainly observed as an improvement in sperm motility parameters [70,85,86,87]. Our results, however, did not show a positive relationship between CAT activity, intracellular ROS, and motility parameters, which would not concur with our previous studies in fresh donkey semen [22] and frozen-thawed donkey and horse sperm [6,17]. Furthermore, in this study, CAT was found to be positively correlated with the proportions of sperm showing an intermediate MMP and negatively with those of sperm exhibiting high membrane lipid disorder, indicating a positive effect of this enzyme on one of the structural sperm parameters. However, these results, together with the aforementioned lower activity of CAT in GFE compared to PFE, would indicate that the activity of CAT in donkey semen could be less relevant as a modulator of H_2_O_2_ than other antioxidant enzymes, such as PON1 or GPX. 

As mentioned above, in addition to SOD, CAT, and GPX, which are considered as fundamental participants in the first line of the cellular antioxidant defense [78], the activity levels of PON1 in donkey SP were also evaluated in this study and were found to be higher than those previously reported in pigs [39,88] and lower than those observed in humans [89]. To the best of our knowledge, this is the first report identifying and quantifying this enzyme in donkey SP. It is well-known that PON1 is an extracellular enzyme associated with high-density lipoproteins (HDL) that has antioxidant and anti-inflammatory properties. It prevents the oxidation of lipoproteins of low (LDL) and high density [88,90], since it binds the cholesterol of the membrane to prevent its oxidation, probably hydrolyzing specific lipid peroxides, such as cholesterol esters and oxidized phospholipids [47,91,92]. In our study, a positive correlation between PON1 activity in SP and sperm with intact plasma membrane (viable) after thawing was found; in addition, intracellular levels of ROS after thawing were negatively correlated with PON1 activity. These data are in agreement with those reported in frozen-thawed pig sperm [39]. In addition, in liquid-stored pig semen, a high PON1 activity in SP was related to an improvement in sperm motility and fertility and a decrease in the generation of intracellular ROS [88]. In humans, while low levels of PON1 activity have been related to an increase in OS and infertility [89,93], high levels of this enzyme have been positively associated with some sperm quality parameters such as concentration, motility, and morphology [89]. Thanks to its antioxidant properties, PON1 protects sperm cells against OS [88,89,91] and exerts a positive influence on sperm quality parameters, as has been observed both in this study and in other species such as pigs [39,88] and humans [89]. This fact, added to the higher activity levels of PON1 in donkey GFE compared to PFE observed in this study and the higher activity of PON1 found in donkey SP when compared to its pig counterpart [39,88], suggests that this enzyme could play a key role in the resilience of donkey sperm to cryopreservation. The measurement of PON1 in donkey SP could thus be a potential freezability biomarker. 

Our results show that the activity levels of non-enzymatic antioxidants (measured in terms of CUPRAC, FRAP, and TEAC) were positively correlated with the percentages of motile and viable sperm after thawing. As expected, their levels, together with total thiol amounts, were negatively correlated with intracellular ROS levels. Results obtained in a recent study in fresh donkey semen were similar to those presented herein, as a positive correlation was not observed between sperm motility and FRAP activity levels nor between sperm motility and total thiols [94]. In addition, our data are in agreement with those reported by Li et al. [39], who observed that the motility of frozen-thawed pig sperm was positively related to higher activity levels of FRAP and TEAC and their viability with higher levels of TEAC and FRAP. It is worth noting that in the case of CUPRAC, our findings differ from those reported by Li et al. [39] in pig semen. Indeed, herein, higher activity levels of CUPRAC, FRAP, and TEAC in GFE compared to PFE were observed, highlighting the involvement of non-enzymatic SP antioxidants in donkey sperm cryotolerance. While FRAP reflects the effect of low-molecular-weight antioxidants, mainly measuring the levels of uric acid, α-tocopherol, and ascorbic acid (AA), CUPRAC and TEAC evaluate the effect of antioxidants that contain sulfhydryl groups in their structure such as thiols (such as reduced glutathione; GSH) and albumin [94,95]. Ascorbic acid and α-tocopherol insert into the membrane structure and are particularly efficient at reducing ROS that interact superficially with membrane components. Specifically, AA reduces nitroxide radicals, and α-tocopherol reduces lipid peroxyl radicals [39]. Furthermore, both are individually sufficient to minimize plasma membrane LPO when present at adequate concentrations [96]. Uric acid is another powerful ROS scavenger that binds to plasma membrane but is not effective in minimizing LPO [39,96]. Reduced glutathione is probably the most important non-enzymatic ROS scavenger and acts mainly via neutralizing ·OH^−^, which is among the most dangerous ROS generated from inactivated H_2_O_2_ [97]. As far as albumin is concerned, in vitro studies have shown that it is an effective antioxidant during LPO, capable of avoiding the damage produced by ·OH^−^, a product of the reaction of H_2_O_2_ and iron [98]. Furthermore, it is known that, among the cationic ligands, copper and iron are very powerful for generating ROS after reaction with oxygen. Free ions of Cu (II) and Fe (II) can interact with H_2_O_2_, leading to the formation of ·OH^−^ through the Fenton reaction [99]. Bound to proteins such as albumin, copper and iron are generally less susceptible to participate in the Fenton reaction, and the ·OH^−^ released by that reaction is mainly directed to proteins such as albumin, avoiding more important targets [99]. 

Because maintaining an adequate balance between ROS and antioxidant levels is essential for optimal sperm function [100], the analysis of SP-OSI, which is considered as a quick, easy, and inexpensive technique to accurately show the oxidant/antioxidant relationship in biological samples [101], was also conducted in this study. To our knowledge, this is the first report in which OSI has been measured in donkey SP in order to evaluate its presumed influence on sperm cryotolerance in mammals. In pig semen, SP-OSI has been related to the ability of sperm to tolerate cooling, showing a positive relationship with the loss of sperm motility and a negative relationship with in vivo fertility of liquid-stored semen [102]. This study found that SP-OSI values were lower in GFE than in PFE. Furthermore, SP-OSI levels were negatively correlated with sperm motility and viability after thawing. These results are in agreement with a previous study carried out in porcine, in which SP-OSI levels were found to be related with the sperm resilience to liquid storage at 17 °C [102]. In addition, SP-OSI levels were negatively correlated with the activity levels of SOD, CUPRAC, FRAP, and TEAC. These findings, together with the positive correlation found between OSI and intracellular levels of ROS, mainly superoxides, would indicate the great relevance of the balance between the cellular antioxidant defense system and the oxidative agents present in sperm, and they would also indicate the importance that the determination of SP-OSI could have to predicting donkey sperm cryotolerance.

Finally, individual variability in the capacity of sperm to withstand cryopreservation has already been described in donkeys [103]. In addition, it has been reported that antioxidant enzymes in SP, such as GPX, CAT, and especially SOD, play a vital function in sperm resilience to freeze-thawing in this species [6]. Our study, in addition to ratifying these previous findings, demonstrated the importance of other SP components with antioxidant properties for donkey sperm cryotolerance, including enzymatic antioxidants, such as PON1, and non-enzymatic components, measured in terms of CUPRAC, TEAC, FRAP and total thiols. The current study also investigated the complex balance between antioxidants and oxidative agents in SP and its influence on donkey sperm freezability, through the assessment of OSI. In relation to this, several efforts have been made to identify markers of frostbite in the whole ejaculate and SP in mammalian species, including donkeys. In this regard and as mentioned above, our study confirms that the activity of SOD in donkey SP could be used as a marker of sperm cryotolerance. In addition, our results also indicate, for the first time in this species, that other enzymatic antioxidants, such as PON1, and non-enzymatic antioxidants, such as CUPRAC, FRAP, and TEAC, as well as OSI in SP could be used as putative cryotolerance biomarkers. Further research addressing whether these SP components play such a role in other species is warranted.

## 5. Conclusions

The present study has shown, for the first time in donkeys, that SP components with antioxidant properties, both enzymatic and non-enzymatic, as well as SP-OSI are related to the sperm resilience to cryopreservation. Specifically, the results indicate that the activity levels of enzymatic (SOD and PON1) and non-enzymatic antioxidants (measured in terms of CUPRAC, FRAP, and TEAC) were higher in the SP of GFE than in that of PFE. An opposite pattern, however, was observed for SP-OSI levels, which were lower in the SP of ejaculates categorized as GFE than in those classified as PFE. These results, therefore, suggest that PON1, SOD, CUPRAC, FRAP, TEAC, and OSI in donkey SP could be used as sperm cryotolerance markers in this species. The measurement of these SP antioxidants and SP-OSI could help us identify those donkey ejaculates that will exhibit worse sperm quality after thawing, as well as those that would require antioxidant supplements to rescue sperm function and survival during conservation. Further research addressing the relationship of these antioxidants and SP-OSI with sperm cryotolerance in other species is warranted.

## Data Availability

All data are contained within the article and Supplementary Materials.

## Supplementary Materials

The following are available online at https://www.mdpi.com/article/10.3390/antiox11020417/s1, Table S1: Mean ± SEM and range of sperm motility parameters after thawing in donkey ejaculates classified as having good (GFE, *n* = 8) or poor freezability (PFE, *n* = 7). Table S2. Mean ± SEM and range of function parameters after thawing in donkey ejaculates classified as having good (GFE, *n* = 8) or poor freezability (PFE, *n* = 7). Table S3: Mean ± SEM and range of the activity levels of enzymatic and non-enzymatic antioxidants in the seminal plasma (SP), as well as the levels of seminal oxidative stress index (OSI) of all donkey ejaculates included in the study.
