# Supplementary material for: Impact of Seminal Plasma Antioxidants on Donkey Sperm Cryotolerance
Source: Antioxidants (Basel). 2022 Feb 18;11(2):417. doi: 10.3390/antiox11020417 (PMC8869541; doi:10.3390/antiox11020417)
Supplement: Supplementary file 1 [file antioxidants-11-00417-s001.zip › antioxidants-1555345-supplementary.pdf]

Table S1. Mean ± SEM and range of the sperm motility parameters after thawing in donkey ejaculates classified as having good (GFE, *n* = 8) or poor freezability (PFE, *n* = 7).

Parameter	GFE		PFE	
	**Mean ± SEM**	**Range**	**Mean ± SEM**	**Range**
TM (%)	48.76 ± 1.58	42.35–53.27	32.29 ± 3.17	16.09–40.14
PM (%)	30.06 ± 1.62	24.88–36.44	17.12 ± 3.82	5.10–30.61
VCL (μm/s)	71.48 ± 6.39	52.71–111.16	67.27 ± 5.68	49.54–94.73
VSL (μm/s)	50.21 ± 6.77	35.35–94.52	48.38 ± 5.65	30.76–70.83
VAP (μm/s)	55.37 ± 7.20	38.36–102.04	52.89 ± 5.82	34.01–75.03
LIN (%)	68.94 ± 2.89	58.54–85.03	70.77 ± 3.27	59.90–80.77
STR (%)	90.48 ± 0.88	87.64–93.66	91.08 ± 0.67	88.88–94.40
WOB (%)	76.13 ± 2.84	65.20–91.80	77.62 ± 3.24	67.40–88.05
ALH (μm)	2.51 ± 0.12	2.08–3.20	2.30 ± 0.12	1.88–2.72
BCF (Hz)	11.95 ± 0.56	9.80–14.39	12.11 ± 0.53	10.28–13.95

TM (%): total motility; PM (%): progressive motility; VCL (μm/s): curvilinear velocity; VSL (μm/s): straight-line velocity; VAP (μm/s): average path velocity; LIN (%): linearity coefficient; STR (%): straightness coefficient; WOB (%): wobble coefficient; ALH (μm): amplitude of lateral head displacement; BCF (Hz): beat-cross frequency.

Table S2. Mean ± SEM and range of the sperm function parameters after thawing in donkey ejaculates classified as having good (GFE, *n* = 8) or poor freezability (PFE, *n* = 7).

Parameter	GFE		PFE	
	**Mean ± SEM**	**Range**	**Mean ± SEM**	**Range**
$SYBR14^+/PI^-$ (%)	48.85 ± 1.23	43.16–55.48	30.43 ± 2.64	20.89–37.40
High MMP (%)	1.81 ± 0.13	1.42–2.36	2.12 ± 0.12	1.72–2.44
Intermediate MMP (%)	39.09 ± 1.95	30.40–46.28	55.64 ± 3.49	42.90–68.52
DCF^+/PI^- (%)	0.07 ± 0.03	0.02–0.27	0.12 ± 0.04	0.04–0.33
E^+/YO-PRO-1^- (%)	0.52 ± 0.13	0.13–1.04	0.91 ± 0.27	0.28–1.97
$Fluo3^+/PI^-$ (%)	8.08 ± 0.93	5.69–14.36	9.92 ± 1.50	4.41–16.11
$M540^+$/YO-PRO-1^- (%)	2.02 ± 0.28	1.12–2.91	1.32 ± 0.20	0.39–1.89

$SYBR14^+$/Propidium iodide $(PI)^-$ (%): sperm with intact plasma membrane (viable sperm); High MMP (%): sperm with high mitochondrial membrane potential; Intermediate MMP (%): sperm with intermediate mitochondrial membrane potential; Dichlorofluorescein $(DCF)^+/PI^-$ (%): viable sperm with high intracellular ROS levels; Ethidium $(E)^+$/YO-PRO-1^- (%): viable sperm with high$\cdot O_2^-$ levels; Fluo3-acetomethoxyester $(Fluo3)^+/PI^-$ (%): viable sperm with high levels of intracellular calcium; Merocyanine 540 $(M540)^+$/YO-PRO-1^- (%): viable sperm with high membrane lipid disorder.

Table S3. Mean ± SEM and range of the levels of enzymatic and non-enzymatic antioxidants in seminal plasma (SP), as well as levels of seminal oxidative stress index (OSI) of all donkey ejaculates included in the study.

Group	Antioxidant	Mean ± SEM	Range
Enzymatic antioxidants	PON1 (IU/L)	0.25 ± 0.04	0.10–0.70
	SOD (IU/mL)	2168.80 ± 216.72	320.00–3784.00
	CAT (IU/mL)	0.26 ± 0.04	0.08–0.48
	GPX (IU/L)	101.59 ± 12.97	10.20–172.00
Non-enzymatic antioxidants	Total thiol (μmol/L)	81.55 ± 8.95	34.30–157.50
	CUPRAC (mmol/L)	1.67 ± 0.14	0.61–2.40
	FRAP (mmol/L)	1.71 ± 0.18	0.34–2.85
	TEAC (mmol/L)	2.30 ± 0.15	0.98–3.03
TOS (μmol/L)		7.41 ± 0.54	2.40–9.30
OSI (arbitrary units)		3.60 ± 0.51	0.88–9.38

PON1 (IU/L): paraoxonase type 1; SOD (IU/mL): superoxide dismutase; CAT (IU/mL): catalase-like; GPX (IU/L): glutathione peroxidase-like; CUPRAC (mmol/L): cupric reducing antioxidant capacity; FRAP (mmol/L): plasma iron-reducing capacity; TEAC (mmol/L): Trolox equivalent antioxidant capacity; TOS (μmol/L): total oxidative status; OSI (arbitrary units): seminal oxidative stress index.
